# Achilles Tendinopathy: From the Basic Science to the Clinic

**DOI:** 10.1155/2017/9534125

**Published:** 2017-04-30

**Authors:** Hong-Yun Li, Youichi Yasui, Seung Hwan Han, Wataru Miyamoto, Ying-Hui Hua

**Affiliations:** ^1^Sports Medicine Center, Fudan University, Department of Sports Medicine and Arthroscopy Surgery, Huashan Hospital, Shanghai, China; ^2^Hospital for Special Surgery, New York, NY, USA; ^3^Department of Orthopaedic Surgery, Yonsei University College of Medicine, Seoul, Republic of Korea; ^4^Department of Orthopaedic Surgery, Teikyo University School of Medicine, Tokyo, Japan

Chronic Achilles tendinopathy is frequent in the individuals who participate in the physical activities, especially running and jumping. This disorder could be subdivided into insertional and noninsertional tendinopathy according to the zone of the pathological disorder.

The essence of tendinopathy is a failed healing response, which includes three different and continuous stages (reactive tendinopathy, tendon disrepair, and degenerative tendinopathy). These tendinopathic lesions affect both collagen matrix and tenocytes. In a recent study, the authors found that the Achilles tendinopathy is harder than asymptomatic tendons on axial-strain sonoelastography, which indicated the decrease of resisting plastic deformation [1]. The histological studies demonstrate disorganization and fragmentation of the collagen, a decrease in collagen fiber diameter and in the overall density of collagen, an increased number of tenocytes and concentration of glycosaminoglycans in the ground substance, and neovascularization.

Metalloproteases (MMPs) are important regulators of extracellular matrix remodeling, and their levels are altered during tendinopathy. Previous studies indicated that MMP-9 and MMP-13 participate in collagen degradation process, whereas MMP-2, MMP-3, and MMP-14 participate in both collagen degradation and collagen remodeling processes. Moreover, difference kinds of growth factors and cytokines such as vascular endothelial growth factor, insulin-like growth factor, and transforming growth factor *β*2 detected increased expression in the Achilles tendon, which could induce neovascularization and stimulate fibroblast and tenocytes proliferation and synthesis of collagen [2].

The reasons of pain in Achilles tendinopathy are complicated. The changes in matrix, vascular, tenocyte, cytokines, neuropeptides and neurotransmitters, and ion channels in the cells are the potential contributors to pain [3]. Moreover, pain could be evoked via nonnociceptive mechanisms through a load detection system, which could be disrupted via local or central dysfunction [3].

Various treatment options have been used for the disorder, which included conservative and surgical treatments.

Conservative treatment was the first line of treatment for Achilles tendinopathy, such as activity modification, orthotics, heel lifts, massage, hot and cold compresses, strengthening exercises, ultrasound, and nonsteroidal anti-inflammatory drugs (NSAID) or corticosteroid injection. Corticosteroid injections may have some benefit in the short term, but adverse effects were reported very high [4]. Therefore, precaution is always advised before the injection of corticosteroid in or around the Achilles tendon. In recent years, the eccentric training program, Extracorporeal Shock Wave Therapy, Deep friction massage, Sclerosing Agents and Aprotinin injection, Electrocoagulation, Prolotherapy (Intratendinous hyperosmolar dextrose), Ultrasound and Low-Level Laser Therapy, Topical Glyceryl Trinitrate, and platelet rich plasma (PRP) injections are introduced to treat Achilles tendinopathy. However, Level I, controlled, randomized studies are still needed to support the effect of these treatments. Among these conservative treatments, the most promising approach is the use of PRP to improve the healing potential of Achilles tendinopathy. However, conflicting results have been reported for PRP injection. The available data do not support the use of PRP as a first-line treatment for chronic tendinopathy. The pharmacological rationale for using PRP in tendinopathy remains unclear. The effect of PRP injection is influenced by many factors, which included the variety of growth factors and cytokines released by the platelets, the time choice for injection, the ability of the factors passing through target tissues, the platelet concentration, and the time of centrifugation. Therefore, a standard should be established for this treatment in future research.

If conservative treatments have no effect, surgical treatments should be considered. The goals of operation are removing degenerative tissue, stimulating tendon healing, and/or augmenting the Achilles tendon with auto- or allografts. Operative treatments seem to be a good option for Achilles tendinopathy patients, when conservative treatment fails. As the lower complication rate, quick recovery, and early return to high-level sports are after surgery, minimally invasive surgery is the primary operative treatment option [5, 6], such as ultrasound + Doppler-guided arthroscopic shaving or minisurgical scraping in degenerative regions with high blood flow and nerves distributions [7]. In the future, long-term follow-ups of more patients and evaluation of tendon thickness and structure integrity are needed.

*Hong-Yun Li* *Hong-Yun Li* *Youichi Yasui* *Youichi Yasui* *Seung Hwan Han* *Seung Hwan Han* *Wataru Miyamoto* *Wataru Miyamoto* *Ying-Hui Hua* *Ying-Hui Hua*
